# Synergistic enhancement of NK cell-mediated cytotoxicity by combination of histone deacetylase inhibitor and ionizing radiation

**DOI:** 10.1186/1748-717X-9-49

**Published:** 2014-02-10

**Authors:** Cheol-Hun Son, Jin-Hee Keum, Kwangmo Yang, Jiho Nam, Mi-Ju Kim, Sun-Hee Kim, Chi-Dug Kang, Sae-Ock Oh, Chi-Dae Kim, You-Soo Park, Jaeho Bae

**Affiliations:** 1 Department of Biochemistry, Pusan National University School of Medicine, Yangsan 626-870, South Korea; 2 Medical Research Center for Ischemic Tissue regeneration, Pusan National University, Busan 609-735, South Korea; 3 Department of Radiation Oncology, Pusan National University Yangsan Hospital, Yangsan 626-770, South Korea; 4 Department of Anatomy, Pusan National University School of Medicine, Yangsan 626-870, South Korea; 5 Department of Pharmacology, Pusan National University School of Medicine, Yangsan 626-870, South Korea; 6 Medical Research Center, Dongnam Institute of Radiological and Medical Sciences, Busan 619-953, South Korea

**Keywords:** NKG2D ligands, HDAC inhibitors, Ionizing radiation, Radioresistance

## Abstract

**Background:**

The overexpression of histone deacetylase (HDAC) and a subsequent decrease in the acetylation levels of nuclear histones are frequently observed in cancer cells. Generally it was accepted that the deacetylation of histones suppressed expression of the attached genes. Therefore, it has been suggested that HDAC might contribute to the survival of cancer cells by altering the NKG2D ligands transcripts. By the way, the translational regulation of NKG2D ligands remaines unclear in cancer cells. It appears the modulation of this unclear mechanism could enhance NKG2D ligand expressions and the susceptibility of cancer cells to NK cells. Previously, it was reported that irradiation can increase the surface expressions of NKG2D ligands on several cancer cell types without increasing the levels of NKG2D ligand transcripts via ataxia telangiectasia mutated and ataxia telangiectasia and Rad3 related (ATM-ATR) pathway, and suggested that radiation therapy might be used to increase the translation of NKG2D ligands.

**Methods:**

Two NSCLC cell lines, that is, A549 and NCI-H23 cells, were used to investigate the combined effects of ionizing radiation and HDAC inhibitors on the expressions of five NKG2D ligands. The mRNA expressions of the NKG2D ligands were quantitated by multiplex reverse transcription-PCR. Surface protein expressions were measured by flow cytometry, and the susceptibilities of cancer cells to NK cells were assayed by time-resolved fluorometry using the DELFIA® EuTDA cytotoxicity kit and by flow cytometry.

**Results:**

The expressions of NKG2D ligands were found to be regulated at the transcription and translation levels. Ionizing radiation and HDAC inhibitors in combination synergistically increased the expressions of NKG2D ligands. Furthermore, treatment with ATM-ATR inhibitors efficiently blocked the increased translations of NKG2D ligands induced by ionizing radiation but did not block the increased ligand translations induced by HDAC inhibitors. The study confirms that increased NKG2D ligand levels by ionizing radiation and HDAC inhibitors could synergistically enhance the susceptibilities of cancer cells to NK-92 cells.

**Conclusions:**

This study suggests that the expressions of NKG2D ligands are regulated in a complex manner at the multilevel of gene expression, and that their expressions can be induced by combinatorial treatments in lung cancer cells.

## Background

It is well known NK cells play a role in immune surveillance for cancer [1] and that their anticancer immunity is controlled by a balance of activating and inhibitory signals [2]. NKG2D is a well characterized immunoreceptor which mediates activating signals on NK cells and T cell subsets, such as, CD8+ and γδT lymphocytes [3]. In humans, eight distinct NKG2D ligands, including MHC class I chain-related gene A/B(MICA/B) and UL16-binding protein 1–6 (ULBP1-6 or RAET1I,H,N,E,G and L), have been described [4]. Furthermore, the induction of NKG2D ligands by several methods, including treatment with anti-cancer drugs, ionizing radiation, heat shock, or proteasomal inhibition, has been proposed as a strategy for eliciting anti-cancer immunity [5-8]. Radiotherapy is a widely used modality to treat cancer; it causes double-strand DNA breaks, and thus, induces cancer cell death. Although it has been reported that ionizing radiation can induce NKG2D ligands on cancer cells by activating the ATM-ATR pathway [9], the precise regulatory mechanism involved is unclear. Of the recently developed anti-cancer agents, HDAC inhibitors have been investigated in treatment of cancers, and it has been reported that several HDAC inhibitors, including suberoylanilide hydroxamic acid (SAHA), tricostatin A (TSA), valproic acid, and PCI-24781, enhance the radiosensitivities of cancer cells [10-13]. Because HDAC inhibitors are known potent inducers of NKG2D ligands on many cancer cells [14,15], it is possible that the induced NKG2D ligands could overcome immune tolerance and make cancer cells sensitive to NK-cell mediated cytotoxicity. Accordingly, we investigated whether ionizing radiation in combination with HDAC inhibitor treatment increases the expressions of NKG2D ligands, and ATM-ATR signaling is involved in this process, and this expressional increases enhances the susceptibility of cancer cell to NK cells.

## Materials and methods

### Cell lines and reagents

Two human non-small cell lung cancer cell lines, A549 and NCI-H23, were used in this study, and were obtained from the Korean Cell Line Bank (Seoul, Korea). These cell lines were maintained in RPMI media supplemented with 10% fetal bovine serum (FBS) (Gibco, Grand Island, NY), 2 mM L-glutamine, 100 μg/ml streptomycin, and 100 U/ml penicillin. The NK-92 cell line was obtained from the American Type Culture Collection (Rockville, MD, USA) and maintained in alpha-Minimum Essential Modified medium supplemented with 12.5% (v/v) fetal bovine serum, 12.5% (v/v) horse serum, 2 mM L-glutamine,0.1 mM 2-mercaptoethanol, 200 U/mL of recombinant human interleukin-2, 100 μg/mL streptomycin, and 100U/mL penicillin. All cells were cultured at 37°C in a humidified atmosphere containing 5% CO_2_.Three HDAC inhibitors, apicidin, suberoylanilide hydroxamic acid (SAHA; vorinostat) and tricostatin A (TSA), two ATM-ATR inhibitors, caffeine, and KU-55933, cycloheximide (CHX) were purchased from Sigma-Aldrich (St. Louis, MO, USA). To irradiate cancer cells, we used a ClinaciX Linear Accelerator (Varian Medical Systems, Inc. Palo Alto, CA, USA) with the assistance of Dr. Jiho Nam (Pusan National University Yangsan Hospital).

### Total RNA extraction and Multiplex Reverse Transcription (RT)-PCR

Total RNA extraction and RT-PCR were performed as previously described [16]. Briefly, total RNA was extracted from cells using the RNeasy® Mini Kit (Qiagen GmbH, Germany). One microgram of extracted total RNA was used to synthesize cDNA using 100 pmol of random primers (Takara, Japan) and 100 U of M-MLV reverse transcriptase (Promega Co., Fitchburg, Wisconsin, USA). The resulting cDNA was used as template for PCR, which was conducted using the QIAGEN® Multiplex PCR Kit (Qiagen GmbH). Seven pairs of primer sets were used to investigate the expressions of the ribosomal protein L19 (RPL19), MICA, MICB, ULBP1-3, and β-actin (ACTB) genes. ACTB and RPL19 were used as a loading control and a degradation marker, respectively. PCR products were stained by ethidium bromide and separated by 2.0% agarose gel electrophoresis, and quantified using image analyzing software (Quantity One; Bio-Rad Laboratories, Inc., Hercules, CA, USA).

### Flow cytometry

To determine the surface expressions of NKG2D ligands on cancer cells, the cells were incubated with mouse anti-MICA, anti-MICB, anti-ULBP1-3 (R&D systems, Minneapolis, MN, USA), anti-HLA-ABC (Clone W6/32, Serotec, Oxford, UK) or the corresponding isotype controls at 10 μg/ml and then incubated with goat anti-mouse-PE conjugated (BD Pharmingen Inc., San Diego, CA., USA). The analysis was performed on a FACS Sort® (Becton Dickinson, Mountain View, CA., USA) using Cell Quest software (Becton Dickinson), and cell surface expressions were quantified using mean fluorescence intensities (MFIs). Relative expression ratios were calculated by dividing treated sample MFI by untreated sample MFI without subtracting the MFI of the appropriate isotype control.

### NK cell-mediated cytotoxicity assay using time-resolved fluorometry

NK cell-mediated cytotoxicity was determined using the DELFIA® EuTDA Cytotoxicity Reagents (PerkinElmer Life Sciences, Waltham, MA, USA), as described previously [17]. Briefly, target cells (1X10^6^ cells/ml) were incubated with freshly prepared 10 μM BATDA (a fluorescence enhancing ligand) in 2 ml of culture medium for 30 min at 37°C, and washed. Next, 100 μl of BATDA-labeled target cells (5,000 cells) were transferred into a round-bottom sterile plate and co-cultured with NK-92 cells for 2 hours at effector/target ratios ranging from 2.5:1 to 10:1. After incubation, 20 μl of supernatant from each well was transferred to the wells of flat-bottom 96 well plates. 180 μl of europium (Eu) solution was then added to form highly fluorescent and stable chelates (EuTDA), and the fluorescences of these chelates were measured by time-resolved fluorometry (Victor3, PerkinElmer). The percent of specific release was calculated using (experimental release – spontaneous release)/(maximum release – spontaneous release) X 100(%). In blocking experiments, blocking anti-NKG2D mAb (R&D Systems) was pre-added to suspensions of NK-92 cells and incubated for 30 min prior to co-cultured with target cells. All experiments were performed in triplicate.

### NK cell-mediated cytotoxicity assay by flow cytometry

Fresh NK cells were obtained from normal healthy donors with informed consent in accordance with the Declaration of Helsinki. Target cells (1X10^5^ cells/ml) were stained using the Vybrant® carboxyfluorescein diacetate, succinimidyl ester (CFSE) Cell Tracer Kit (Invitrogen, Eugene, OR, USA) and incubated with NK-92 cells or freshly isolated NK cells at selected effector/target ratios for 2 hours in 5 ml round-bottomed tubes. These co-cultured target cells and NK cells were then stained with 1 μg/ml propidium iodide (Sigma-Aldrich). The assay was performed on FACS Sort® (Becton Dickinson) by acquiring 3,000 target cells. The percent if specific release was calculated by the number of PI^+^&CFSE^+^ cells/3,000 X 100 (%). All experiments were performed in triplicate.

### Statistical analysis

To evaluate alterations in gene expression, the gene expressions in treated cells were divided by those in untreated controls (mean fold) and standard errors (SE) were calculated. To compare groups, we used the paired Student’s *t*-test. Statistical significance was accepted for p values < 0.05.

## Results

### HDAC inhibitors increase the mRNA expressions of NKG2D ligands but ionizing radiation minimally alters these expressions in A549 cells

**Figure 1:**
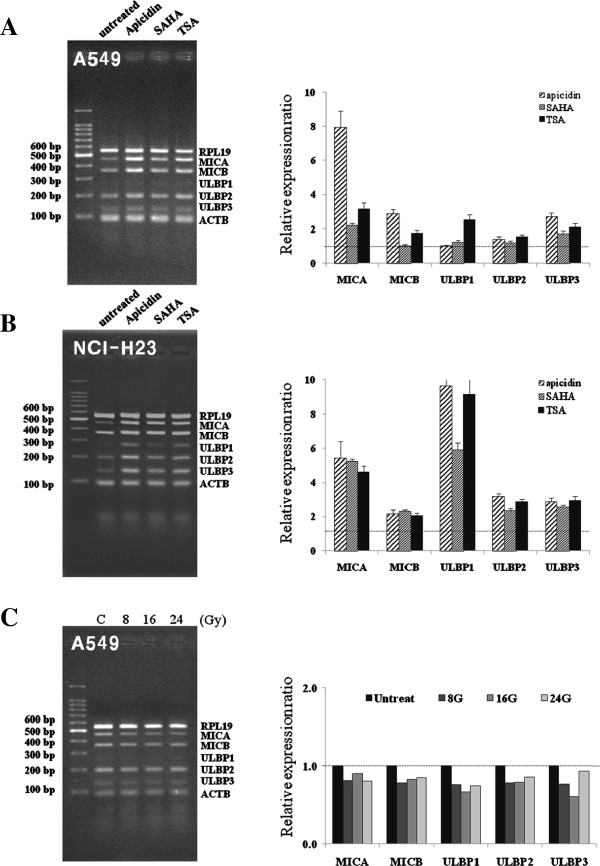
**Analysis of the expressions of NKG2D ligands after treating lung cancer cells with HDAC inhibitors or ionizing radiation.** Multiplex RT-PCR analysis was performed to determine the mRNA expressions of NKG2D ligands. A549 and NCI-H23 cells were treated with 125 ng/ml apicidin, 2.5 μ M SAHA, or 250 nM TSA **(A, B)** for 12 hours. A549 cells were irradiated with the indicated doses and allowed to recover for 24 hours **(C)**. Amplified DNA was quantitated using Quantity-one software (Bio-rad). All experiments were performed at least three times, and representative results are shown. MICA/B: MHC class I chain-related molecules A/B, ULBP1/2/3: UL16-binding proteins 1/2/3, ACTB: -actin, RPL19: ribosomal protein19.

The mRNA expressions of NKG2D ligands, MICA/B and ULBP1-3, were analyzed after treating A549 cells with three HDAC inhibitors, that is, 125 ng/ml apicidin, 2.5 μM SAHA, or 250 nM TSA, for 12 hours. Levels of NKG2D ligands were significantly increased after treatment with two HDAC inhibitors, apicidin and TSA, in A549 cells (Figure 1A). SAHA increased the mRNA levels of only MICA and ULBP3. Although ULBP1 tend to increase, alterations of ULBP1 transcript levels were statistically insignificant in apicidin or SAHA treated A549 cells. The mRNA levels of the five NKG2D ligands were also significantly increased after treatment with the three HDAC inhibitors in NCI-H23 cells (Figure 1B). However, ionizing radiation did not significantly change the mRNA levels of the five NKG2D ligands in A549 cells (Figure 1C). Although previous reports have shown that ionizing radiation significantly increases the mRNA and protein expressions of NKG2D ligands in several cancer cells, including NCI-H23 cells [6], it was thought that the induction of NKG2D ligands were different from individual cancer cells. The reason for this lack of responsive in A549 cells to ionizing radiation is not clear.

### Combination of HDAC inhibitors and ionizing radiation prominently increases the surface expressions of NKG2D ligands

**Figure 2:**
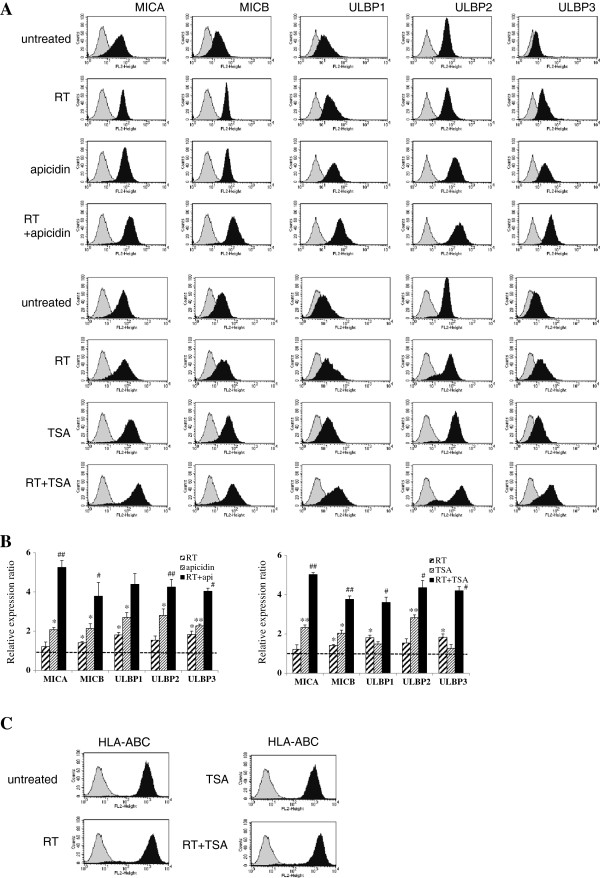
**Cell surface expressions of NKG2D ligands after treating irradiated A549 cells with HDAC inhibitors.** NKG2D ligand cell surface expressions were analyzed by flow cytometry using specific mAbs against NKG2D ligands. A549 cells were irradiated with 8 Gy, allowed to recover for 6 hours, and then treated with or without 250nM TSA or 125 ng/ml apicidin. The cells were then incubated for 18 hours. In the figure, filled gray represents the isotype control and filled black represents the treated group **(A)**. All experiments performed independently in triplicate and significant differences between the untreated control and treated cells are presented as * (p < 0.05) or ** (p < 0.01). Ratios of mean fluorescence intensities (MFI) obtained from treated cells and untreated control are shown **(B)**. The further inductions of NKG2D ligands by combination treatment as compared with treatment with HDAC inhibitors only are marked # (p < 0.05) or ## (p < 0.01). Changes in the surface protein expressions of HLA-A/B/C were analyzed after ionizing radiation or TSA treatment **(C)**.

Flow cytometric analysis showed that the cell surface protein levels of the five NKG2D ligands were significantly increased after treatment with 125 ng/ml apicidin and 250 nM TSA for 18 hours following increased transcripts in A549 cells. Although alterations in the protein levels of MICA and ULBP2 were not significant, 8Gy of irradiation and 24 hour-recovery incubation increased the surface protein expressions of NKG2D ligands despite little change in their mRNA levels. Relative expression ratios were calculated by dividing the MFI ratios of treated samples by those of untreated samples. These results suggested that the induction of NKG2D ligands was achieved at different levels of gene expression by HDAC inhibitors and ionizing radiation differentially. When HDAC inhibitors plus ionizing radiation were administered to A549 cells, surface NKG2D ligand protein levels were further increased as compared with cells treated with HADC inhibitors or ionizing radiation only (Figure 2A-B). We considered that this further induction of NKG2D ligands might be due to the promotion of transcription and post-transcription processes. On the other hand, the surface expression of HLA-ABC, which inhibits NK cells, was slightly increased after ionizing radiation but was blocked by HDAC inhibitor (Figure 2C).

### The susceptibility of A549 cells to NK cells is synergistically increased by HDAC inhibitors treatment and ionizing radiation in combination

**Figure 3:**
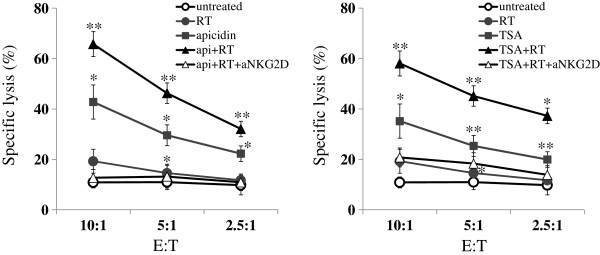
**Increased susceptibility of A549 cells to the cytolytic activity of NK-92 cells following HDAC inhibitor and ionizing radiation co-treatment.** A549 cells were not treated (open circle), irradiated with 8 Gy (filled circle), treated with 125 ng/ml apicidin or 250 nM TSA (filled square), or co-treated with after ionizing radiation plus HDAC inhibitors (apicidin or TSA; filled triangle). Cells were co-cultured with NK-92 cells at the indicated effector/target ratio. To determine the specificity of NKG2D-mediated cytolysis, NK-92 cells were pre-incubated with blocking mAb against NKG2D (open triangle). All experiments were performed in triplicate and significant differences between NK cell-mediated lyses of untreated and treated cells were accepted for *P* values of < 0.05.(*; *p* < 0.05, **; *p* < 0.01).

To investigate whether treatment with HDAC inhibitor and irradiation increase the NK cell-mediated lysis of cancer cells, cytotoxicity assays were performed using DELFIA® EuTDA Cytotoxicity Reagents. The susceptibility of A549 cells to NK cell-mediated lysis was further increased by treatment with ionizing radiation plus TSA, and this was prevented by adding blocking mAb against NKG2D prior to the assay (Figure 3). Although individual and experimental variations were high, cytotoxic assays using NK cells derived from three healthy donors showed that the recognition of A549 cells by freshly isolated NK cells tended to be further increased by combination treatment (Additional file 1). These results might indicate that the observed increase in NK cell-mediated lysis of A549 cells was due to the increased expressions of NKG2D ligands. However, despite significant induction of NKG2D ligands by ionizing radiation, ionizing radiation did not significantly increased the susceptibility of A549 cells to NK cells. Although the reason is unclear, it is possible that increased levels of inhibitory molecules (HLA-A/B/C) in A549 cells might have affected their recognition to NK cells. However this adverse effect of ionizing radiation was prevented by HDAC inhibitors.

### Single use of ATM-ATR inhibitors do not significantly change the expressions of NKG2D ligands at the mRNA level

**Figure 4:**
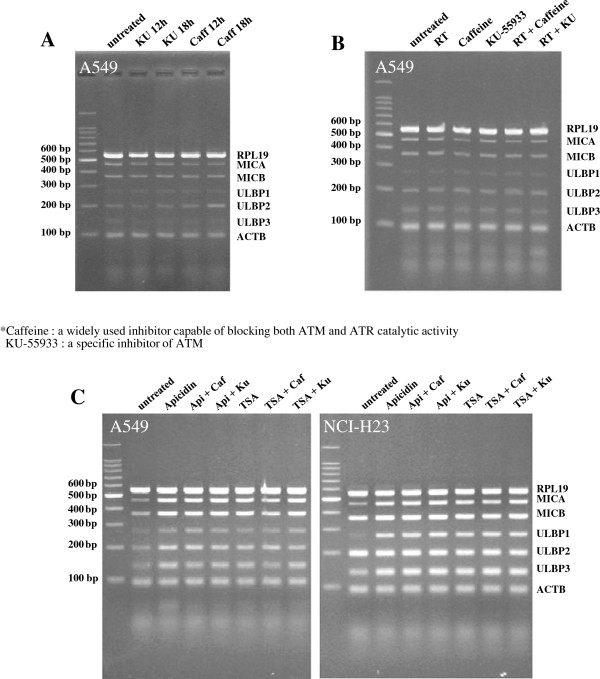
**Effects of ATM-ATR inhibitors on the transcriptions of NKG2D ligands in lung cancer cells.** A549 cells were unaffected by KU-55933 or caffeine **(A)**, though caffeine marginally suppressed MICA transcription. Radiation or radiation plus ATM-ATR inhibitors did not affect NKG2D ligand transcription in A549 cells **(B)**. The induced transcription of NKG2D ligands by HDAC inhibitors was not blocked by ATM-ATR inhibitors in A549 or NCI-H23 cells **(C)**.

It was demonstrated that ionizing radiation increases the NKG2D ligands through ATM-ATR signaling [9,18]. To investigate the role of ATM-ATR signaling, a selective ATM-ATR inhibitor (KU-55933) and a broad nonspecific inhibitor (caffeine) were used. Although mRNA of MICA tended to decrease by treatment with caffeine, treatment with KU-55933 or caffeine did not affect the expressions of NKG2D ligands at the mRNA level (Figure 4A). Furthermore, transcriptional alteration of NKG2D ligands were not observed even after their combination with ionizing radiation or HDAC inhibitors (Figure 4B, C).

### ATM-ATR inhibitors block the inductions of surface proteins of NKG2D ligands by ionizing radiation but not their inductions by TSA

**Figure 5:**
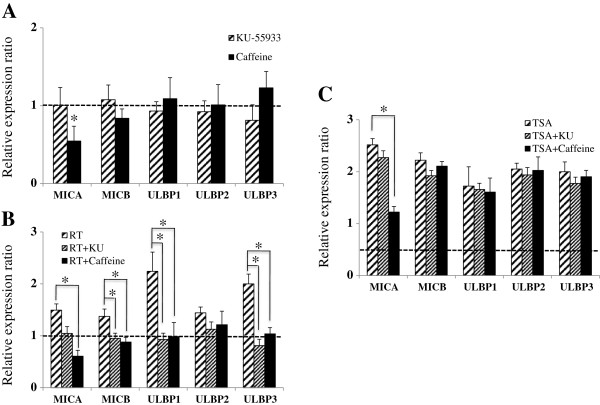
**Effects of ATM-ATR inhibitors on the surface expressions of NKG2D ligands in A549 cells.** The surface protein expressions of NKG2D ligands on A549 cells, MICA, were unaffected by KU-55933 or caffeine **(A)**; caffeine significantly decreased the surface expression of MICA. The induction of NKG2D ligands surface protein by ionizing radiation was blocked by ATM-ATR inhibitors in A549 cells, although difference of ULBP2 was not significant. **(B)**. The induction of the surface expressions of NKG2D ligands by HDAC inhibitors was not blocked by ATM-ATR inhibitors in A549 cells, with the exception of MICA in cells treated with caffeine **(C)**.

KU-55933 or caffeine did not affect the surface expressions of NKG2D ligands, except MICA (Figure 5A). Caffeine essentially suppressed the expression of MICA at surface protein level via an undefined mechanism. The inductions of NKG2D ligands by ionizing radiation were effectively blocked by ATM-ATR inhibitors (Figure 5B). However, induction by TSA was not blocked by ATM-ATR inhibitors, except that of MICA (Figure 5C). It was suggested that the ATM-ATR pathway might have role in the effects of ionizing radiation but be independent on the expression of NKG2D ligands in inhibition of HDAC.

### Protein synthesis inhibitor efficiently suppressed the inductions of NKG2D ligands by TSA

**Figure 6:**
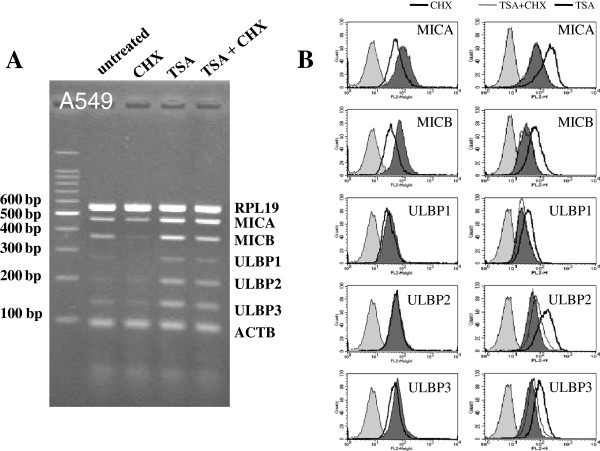
**Induction of surface NKG2D ligands by TSA treatment was blocked by CHX in A549 cells.** A549 cells were treated with CHX (1 μg/ml) and 250 nM TSA for 18 hours, and single treatment of CHX reduced the transcription of NKG2D ligands **(A)**. However, the transcriptional induction of NKG2D ligands by TSA was not blocked by co-treatment with CHX **(B)**. Although the suppressions of ULBP1-3 were not statistically significant, CHX effectively blocked the surface expression of MICA and MICB by TSA. Filled gray, filled dark gray, thick line and thin line represent isotype control, untreated control, CHX or TSA single treatments, and TSA plus CHX combined treatment, respectively. Significant differences between untreated control and treated cells are presented as * (*p* < 0.05) or ** (*p* < 0.01).

Cycloheximide (CHX; a protein synthesis inhibitor) was used to discriminate the level of NKG2D ligand regulation. After CHX was administered, the mRNA and surface protein levels of NKG2D ligands decreased regardless of treatment type (Figure 6A, 6B left panel, Additional file 2). Although the mechanism responsible for the suppressive effect of CHX on the expressions of NKG2D ligands was not determined, it was considered that the synthesis of essential enzymes associated with gene transcription, including RNA polymerases, might have been partially inhibited by CHX. However, TSA did overcome the suppressive effect of CHX on the transcriptions of NKG2D ligands. The surface inductions of NKG2D ligands by TSA was blocked by co-treatment with CHX (Figure 6B right panel), which were not affected by co-treatment with ATM-ATR inhibitors (Figure 4C, 5C). Based on these results, we suppose that the post-transcriptional regulation of NKG2D ligands through ATM-ATR signaling might occur at prior step of protein synthesis via a mechanism independent of TSA.

## Discussion

NK cells are remarkably cytotoxic to many different cancer cells *in vitro* and *in vivo*. Moreover, NK cell cytotoxicity is controlled by a signaling balance involving the activations and inhibitions of cell surface receptors. Accordingly, the inductions of NKG2Dligands, which are activating ligands in cancer cells, may provide an attractive means of promoting cancer cell recognition by NK cells [19]. It is generally accepted that NKG2D surface protein expressions are restricted in normal cells despite of presence of their transcripts [20]. In our previous experiments, the mRNA levels and surface protein expressions of NKG2D levels were often discordant. In fact, some cancer cells exhibited increased NKG2D ligand surface expressions but no change in mRNA levels after irradiation, which suggests the expressions of NKG2D ligands are strictly regulated at transcriptional and post-transcriptional levels.

A variety of stresses, such as, heat shock and exposure to hydrogen peroxide, DNA damaging agents, or viral or bacterial infection can increase the expressions of MICA/B [8,9,21,22], although the mechanisms involved remain unclear. Recently, it was reported that chromatin remodeling agents, such as, inhibitors of DNA methyltransferase or nuclear histone deacetylase increase the expressions of many proapoptotic or tumor suppressor genes, and thus can induce the growth arrest, differentiation, and apoptosis of cancer cells [23-25]. In addition, the expressions of NKG2D ligands have been reported to be upregulated at the transcription level in some cancer cells [14,15,26]. On the other hand, DNA damaging agents usually increase the surface expressions of NKG2D ligands at post-transcriptional level in macrophage through ATM-ATR signaling [18]. Therefore, to further increase the expression of NKG2D ligands in cancer cells, we co-treated cells with ionizing radiation and HDAC inhibitors. We presumed that HDAC inhibitors increase the transcription and ionizing radiation increases the translation of NKG2D ligands via different mechanisms.

The lung adenocarcinoma cell line A549 is resistant to ionizing radiation and to cell-mediated killing [27,28]. In the present study, we found that ionizing radiation did not significantly increase NKG2D ligand transcript expression in this cell line, but it did increase their protein levels. On the other hand, ionizing radiation significantly increased the expressions of NKG2D ligands at the mRNA and protein levels in NCI-H23 cells (a radiosensitive lung adenocarcinoma cell line). Although we did not investigate the reason for the different responses to ionizing radiation of these two lung cancer cells, it has been shown that they exhibit different p53 activities [29]. Accordingly, our findings suggest that ionizing radiation and HDAC inhibitor co-treatment increase NKG2D ligand expression and enhance the susceptibility of cancer cells to NK-92 cells and freshly isolated NK cells (Figure 3 and Additional file 1). To examine the effects of ionizing radiation and of HDAC inhibitor treatment separately, we inhibited ATM-ATR signaling, which is activated by ionizing radiation and increased the NKG2D ligand expression [9]. We choose two ATM-ATR inhibitors, that is, caffeine and KU-55933, and pretreated cancer cells with these inhibitors prior to administering ionizing radiation or HDAC inhibitors. ATM-ATR inhibitors effectively blocked the induction of NKG2D ligands by ionizing radiation. However, not by HDAC inhibitors except MICA. These findings show that ionizing radiation and HDAC inhibitors differentially affect the ATM-ATR pathway and NKG2D ligand expression. More specifically, caffeine suppressed the expression of MICA at the surface protein level (Figure 5), and although the mechanism of MICA down-regulation by caffeine is not known, it has been reported that MICA transcription is reduced via the inhibitions of PI3K and PKC, which regulators of MICA transcription [17,30] and caffeine might affect the PI3K and PKC activities. CHX (an inhibitor of protein synthesis) treatment effectively blocked NKG2D ligand induction by HDAC inhibitors. We are of the opinion that ATM-ATR signaling probably does not increase the protein synthesis of NKG2D ligands but rather promotes their translation at a prior step of protein synthesis.

In previous studies, post-transcriptional and -translational regulations were found to be involved in the control of the surface protein levels of NKG2D ligands, and discrepancies between the transcription and surface NKG2D expressions of ligands have often been described [31-33]. We found that co-treatment with ionizing radiation and HDAC inhibitors further increases NKG2D ligand expressions via independent mechanisms in lung cancer cells. BecauseA549 cells did not response to ionizing radiation with respect to the transcriptions of NKG2D ligands and these cells were essentially less susceptible to NK cells, it would appear in this cell-line that by ionizing radiation in the inductions of the surface protein expressions of NKG2D ligands were limited. Although radioresistant lung cancer cells, such as, A549 cells, survive even high-doses irradiation, it appears that co-treatment with ionizing radiation and HDAC inhibitors might be helpful.

## Conclusions

This study suggests NKG2D ligands are regulated in a complex, multi-level manner and that they can be induced by ionizing radiation plus HDAC inhibitors in lung cancer cells. We believe that such combination therapies offer an attractive means of improving the efficacy of NK cell-based cancer immunotherapy in patients with radioresistant cancer.

## Competing interests

The authors have no conflict of interest to declare.

## Authors’ contributions

CHS and JHK carried out the studies and participated in experiments. SHK, CDK, SOO and CDK participated in the study design and helped to draft the manuscript. KY and JN conducted the irradiation experiments. MJK performed the statistical analysis. All authors read and approved the final manuscript.

## Authors’ information

Co-first authors: Cheol-Hun Son and Jin-Hee Keum.

## Supplementary Material

Additional file 1 **Increased susceptibility of A549 cells to the cytolytic activities of fresh isolated NK cells after treatment with ionizing radiation plus TSA.** A549 cells were co-cultured with NK-92 cells or freshly isolated NK cells, the latter of which were obtained from three healthy donors after obtaining informed consent, at the indicated effector/target ratio. The cytotoxicity assay was performed by using flow cytometry and representative results were shown (A). Cytotoxicity assay results were shown as marks (B). untreated (open circle), or irradiated with 8 Gy (filled circle), with 250 nM TSA (filled square), or with RT plus TSA (filled triangle). All experiments were performed in triplicate and significant differences between NK cell-mediated lyses of untreated and treated cells were accepted for *P* values of <0.05.(* ;*P* < 0.05). Click here for file

Additional file 2 **Blockade of the radiation-induced surface expressions of NKG2D ligands by CHX in A549 cells.** A549 cells were irradiated with 8 Gy, allowed to recover for 6 hours, and then treated with or without 250nM TSA or 125 ng/ml apicidin. Cells were then incubated for 18 hours. Filled gray represents the isotype control, filled dark gray the untreated control, the thick line represents irradiated cells, and the thin line represents ionizing radiation plus CHX treated cells. Click here for file
