# Supplementary figures and images for: Synergistic enhancement of NK cell-mediated cytotoxicity by combination of histone deacetylase inhibitor and ionizing radiation
Source: Radiat Oncol. 2014 Feb 10;9:49. doi: 10.1186/1748-717X-9-49 (PMC3923253; doi:10.1186/1748-717X-9-49)

## Slide 1
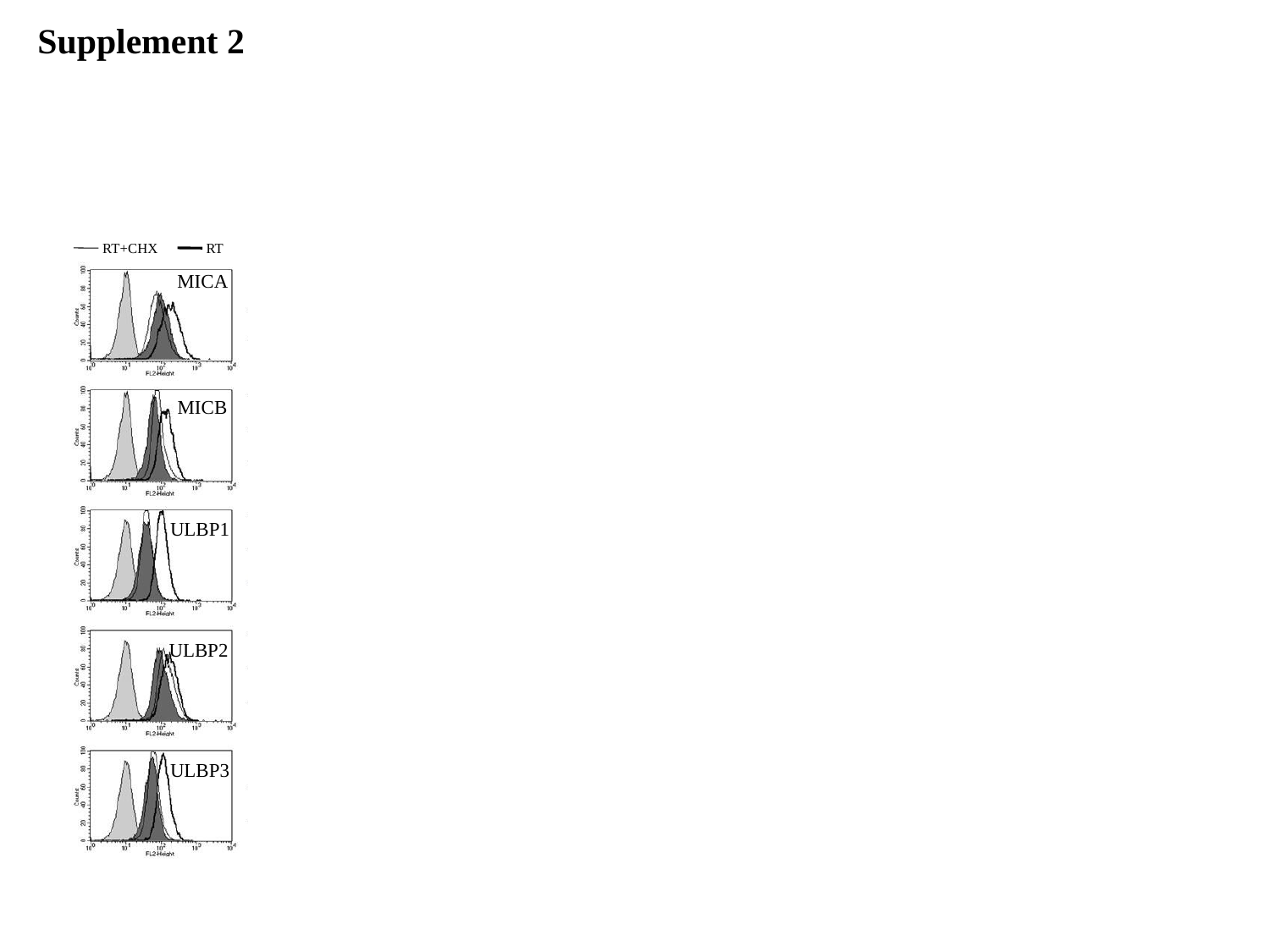

Supplement 2
RT+CHX
RT
MICA
MICB
ULBP1
ULBP2
ULBP3

Supplement: Additional file 1 — Increased susceptibility of A549 cells to the cytolytic activities of fresh isolated NK cells after treatment with ionizing radiation plus TSA. A549 cells were co-cultured with NK-92 cells or freshly isolated NK cells, the latter of which were obtained from three healthy donors after obtaining informed consent, at the indicated effector/target ratio. The cytotoxicity assay was performed by using flow cytometry and representative results were shown (A). Cytotoxicity assay results were shown as marks (B). untreated (open circle), or irradiated with 8 Gy (filled circle), with 250 nM TSA (filled square), or with RT plus TSA (filled triangle). All experiments were performed in triplicate and significant differences between NK cell-mediated lyses of untreated and treated cells were accepted for P values of <0.05.(* ;P < 0.05). [file 1748-717X-9-49-S1.pptx]

## Slide 1
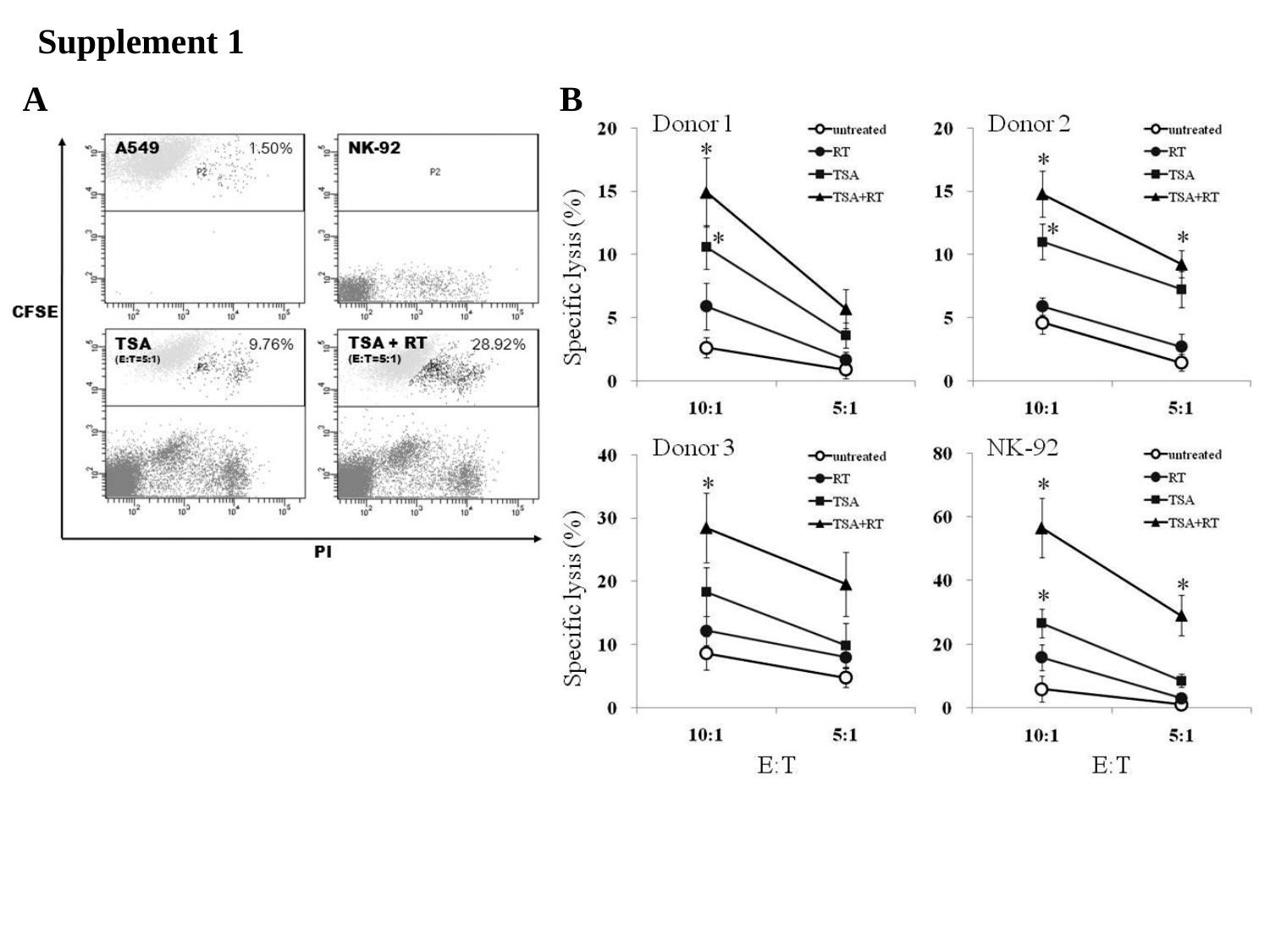

Supplement 1
A
B

Supplement: Additional file 2 — Blockade of the radiation-induced surface expressions of NKG2D ligands by CHX in A549 cells. A549 cells were irradiated with 8 Gy, allowed to recover for 6 hours, and then treated with or without 250nM TSA or 125 ng/ml apicidin. Cells were then incubated for 18 hours. Filled gray represents the isotype control, filled dark gray the untreated control, the thick line represents irradiated cells, and the thin line represents ionizing radiation plus CHX treated cells. [file 1748-717X-9-49-S2.pptx]
